# Risk factors for postoperative morbidity, prolonged length of stay and hospital readmission after appendectomy for acute appendicitis

**DOI:** 10.1007/s00068-023-02225-9

**Published:** 2023-01-28

**Authors:** Bruno Leonardo Bancke Laverde, Matthias Maak, Melanie Langheinrich, Stephan Kersting, Axel Denz, Christian Krautz, Georg Ferdinand Weber, Robert Grützmann, Maximilian Brunner

**Affiliations:** 1 Department of General and Visceral Surgery, Friedrich-Alexander-University Erlangen-Nürnberg (FAU), Krankenhausstraße 12, 91054 Erlangen, Germany; 2 Department of General, Visceral, Thoracic and Vascular Surgery, University Greifswald, Ferdinand-Sauerbruch-Straße, 17475 Greifswald, Germany

**Keywords:** Acute appendicitis, Appendectomy, Risk factors, Morbidity, Readmission

## Abstract

**Purpose:**

The aim of the present study was to identify risk factors associated with postoperative morbidity and major morbidity, with a prolonged length of hospital stay and with the need of readmission in patients undergoing appendectomy due to acute appendicitis.

**Methods:**

We performed a retrospective analysis of 1638 adult patients who underwent emergency appendectomy for preoperatively suspected acute appendicitis from 2010 to 2020 at the University Hospital Erlangen. Data including patient demographics, pre-, intra-, and postoperative findings were collected and compared between different outcome groups (morbidity, major morbidity, prolonged length of postoperative hospital stay (LOS) and readmission) from those patients with verified acute appendicitis (*n* = 1570).

**Results:**

Rate of negative appendectomies was 4%. In patients with verified acute appendicitis, morbidity, major morbidity and readmission occurred in 6%, 3% and 2%, respectively. Mean LOS was 3.9 days. Independent risk factors for morbidity were higher age, higher preoperative WBC-count and CRP, lower preoperative hemoglobin, longer time to surgery and longer duration of surgery. As independent risk factors for major morbidity could be identified higher age, higher preoperative CRP, lower preoperative hemoglobin and longer time to surgery. Eight parameters were independent risk factors for a prolonged LOS: higher age, higher preoperative WBC-count and CRP, lower preoperative hemoglobin, need for conversion, longer surgery duration, presence of intraoperative complicated appendicitis and of postoperative morbidity. Presence of malignancy and higher preoperative WBC-count were independent risk factors for readmission.

**Conclusion:**

Among patients undergoing appendectomy for acute appendicitis, there are relevant risk factors predicting postoperative complications, prolonged hospital stays and readmission. Patients with the presence of the identified risk factors should receive special attention in the postoperative course and may benefit from a more individualized therapy.

**Supplementary Information:**

The online version contains supplementary material available at 10.1007/s00068-023-02225-9.

## Introduction

Acute appendicitis is one of the most common diseases in visceral surgery, worldwide. Analyses of the last years show a consistently high incidence in western countries. Moreover, data from newly industrialized countries suggests that appendicitis is rising rapidly in these regions. During the twenty-first century, the incidence of acute appendicitis in Western Europe was 151/100,000 person-years [1]. In Germany, this rate was slightly lower (123/100,000 person-years in 2017) [2].

The treatment of acute appendicitis is subject of a continuing debate about the best practice of therapeutic management. There are several studies, which suggest a non-operative management of acute appendicitis by antibiotic therapy [3–7]. Proponents of conservative treatment argue with the risk of surgical complications in favor of conservative therapy.

However, until now prompt appendectomy may be still the therapy of choice for most patients with acute appendicitis [8, 9]. Moreover, previous studies show moderate morbidity rates after appendectomy between six and nine percent [10–14].

Nevertheless, knowledge of risk factors, that could affect negatively the surgical outcome, can help to improve the quality of treatment through differentiated treatment and individual postoperative measures for at-risk patients. Perioperative complication rate as well as length of postoperative stay and readmission rate represent important universal indicators of surgical treatment quality.

The primary objective of the present study was to identify risk factors associated with the development of morbidity and major morbidity, with a prolonged length of hospital stay and with the need of readmission in patients undergoing appendectomy due to acute appendicitis.

## Methods

This retrospective analysis includes 1638 consecutive patients (age ≥ 18 years), who underwent an emergency appendectomy for preoperatively suspected acute appendicitis at the Department of General and Visceral Surgery of the University Hospital of Erlangen between January 2010 and December 2020. Patients with one of the following criteria were excluded: (1) patients with age younger than 18 years; (2) appendectomies without preoperatively suspected acute appendicitis.

Patients were stratified into two groups: (1) patients with intraoperatively or histopathologically verified acute appendicitis and (2) patients with negative appendectomies. Postoperative outcome parameters (morbidity, major morbidity, length of hospital stays and readmission) were compared between these two groups.

Further analysis was performed using only patients with intraoperatively or histopathologically verified acute appendicitis. Therefore, data about patients demographics, comorbidities, preoperative findings (blood results, ultrasound, CT scan), intraoperative findings, histopathological findings and postoperative course were obtained and analyzed. The time interval between the first clinical examination of the patients and the first incision for the operation was defined as the time to appendectomy. Gangrenous or perforated appendicitis and/or presence of perithyphilitic abscess were defined as complicated appendicitis.

Primary aim of this study was to identify risk factors for morbidity, major morbidity, prolonged length of hospital stays and readmission after appendectomy for acute appendicitis. Therefore, we stratified our acute appendicitis study cohort according to these four parameters into comparison groups: No postoperative morbidity versus postoperative morbidity, no major postoperative morbidity versus major postoperative morbidity, no prolonged length of hospital stay (LOS) versus prolonged LOS and no readmission versus readmission. Morbidity was defined as any deviation from the normal postoperative course and was classified according to the Clavien–Dindo classification [15]. Morbidity ≥ grade III according to Clavien–Dindo was considered as major morbidity. A postoperative hospital stay > 5 days was recognized as prolonged length of hospital stay (LOS). All readmissions within three months after surgery, which were related to appendectomy, were considered.

This study was approved by the Ethics Committee of FAU Erlangen (22-157-Br).

### Diagnostic and therapeutic algorithm of patients with acute appendicitis

At first admission, all patients received a blood test including hemogram, inflammatory and coagulation parameters as well as an abdominal ultrasound preoperatively. If diagnosis was still unclear an abdominal CT-scan was indicated. Intraoperative findings were documented by the surgeons in their surgical report. The intraoperative collection of material for microbiological examination was made depending on the surgeon’s decision. All appendectomy specimens were examined histopathologically. In the postoperative course, blood examinations were carried out depending on the clinical status of the patients.

### Statistical analysis

Statistical analyses were performed with SPSS Statistic (Version 28.0, IBM). Comparisons of ordinal and metric data were calculated with Mann–Whitney *U* test or Student’s *t* test. For categorical data, the chi-square test was used. Statistical significance was set a *p* < 0.05. Multivariate analysis was performed with in univariate analysis identified risk factors for at least one of the outcome parameter (morbidity, major morbidity, prolonged LOS or readmission). Parameter with incomplete data and postoperative parameters, which represent sequelae and not risk factors of outcome parameters were excluded from the multivariate analysis. Cutoffs for metric risk factors were calculated using ROC analysis. Independent risk factors in the multivariate analysis were included in analysis of risk factor score.

## Results

A total of 1638 patients, who underwent emergency appendectomy during study period, were included.

### Negative appendectomy vs. appendectomy for acute appendicitis

Negative appendectomies were performed in 68 patients (4%). Patients with negative appendectomy had a significantly shorter length of postoperative stay compared to those with verified acute appendicitis (3.4 vs. 3.9 days, *p* = 0.003). Morbidity, major morbidity and readmission occurred in 3%, 3% and 0%, respectively, and did not differ compared to patients with appendicitis.

### Outcome parameter after appendectomy for acute appendicitis

In 1570 patients, acute appendicitis was verified intraoperatively and/or histopathologically. Mean age of these patients was 39 ± 17 years. 48% of patients were female. In 99 patients (6%) postoperative morbidity occurred. The most common complications were wound infections (19%), followed by intra-abdominal abscesses (13%) and non-surgical cardiopulmonary complications (12%) (Table 1). 49% of patients with postoperative complications suffered from minor morbidity (Clavien–Dindo I and II), whereas 51% had major morbidity (Clavien–Dindo III to V). Two cases of in-hospital-mortality (0.13%) were observed: One 80-year-old man with myelodysplastic syndrome and advanced appendicitis underwent an appendectomy by conversion and developed postoperative intestinal ischemia. The second patient was a 60-year-old male, who underwent an uncomplicated open appendectomy and who was found dead on the 2nd postoperative day after an uncomplicated postoperative course. The cause of death remained unclear as no autopsy was performed. Mean length of postoperative hospital stay (LOS) amount 3.9 ± 4.5 days. In 21 patients (1%), a reoperation was necessary. There were 30 patients requiring appendectomy-related readmission (2%) (Table 1).

**Table 1:** Outcome parameter (morbidity, length of hospital stay and hospital readmission) after appendectomy for acute appendicitis (*n* = 1570) and negative appendicitis (*n* = 68)

	Outcome parameter	Intraoperative and/or pathological appendicitis	Negative appendectomies	*p* value
Number		1570 (96)	68 (4)	
In-hospital	Clavien–Dindo			0.317
	0	1471 (94)	66 (97)	
	I	25 (2)	0 (0)	
	II	24 (1)	0 (0)	
	III	38 (2)	2 (3)	
	IV	10 (1)	0 (0)	
	V	2 (0)	0 (0)	
	Cause for morbidity			–
	Surgical causes			
	Wound infection	19 (19)	0 (0)	
	Intra-abdominal abscess	13 (13)	0 (0)	
	Paralysis/ileus	6 (6)	0 (0)	
	Bleeding	4 (4)	0 (0)	
	Appendiceal stump insufficiency	0 (0)	0 (0)	
	Non-surgical causes	12 (12)	1 (50)	
	Cardio-pulmonary complication	12 (12)	1 (50)	
	Uro-genital complication	7 (7)	0 (0)	
	Others	38 (38)	1 (50)	
	Re-surgery	21 (1)	1 (1)	1.000
	Length of postoperative hospital stay (in days)	3.9 ± 4.5	3.4 ± 3.6	**0.003**
After discharge	Appendectomy-related readmission	30 (2)	0 (0)	0.269
	Reasons for readmission		–	–
	Paralysis/ileus	9 (30)		
	Intra-abdominal abscess	5 (17)		
	Malignancy	5 (17)		
	Wound infection	4 (13)		
	Abdominal pain	4 (13)		
	Others	3 (10)		

Significant p values are in bold

### Baseline patient characteristics of patients with acute appendicitis

Demographics characteristic of patients stratified to the subgroups morbidity, major morbidity, prolonged LOS and readmission are shown in Table 2. Risk factors for at least one of the outcome parameter were age, BMI, ASA, Diabetes. Gender, smoking, Crohn’s disease and ulcerative colitis did not differ between the groups.

**Table 2:** Baseline patient characteristics and preoperative findings

	No morbidity	Morbidity	p value	No major morbidity	Major morbidity	*p* value	No prolonged LOS	Prolonged LOS	*p* value	No	Re-admission	*p* value
							(≤ 5 days)	(> 5 days)		readmission		
Number	1471	99		1520	50		1361	209		1540	30	
Age (years)	38 ± 17	57 ± 18	**< 0.001**	39 ± 17	58 ± 18	**< 0.001**	37 ± 16	54 ± 19	** < 0.001**	39 ± 17	41 ± 17	0.357
Gender			1.000			0.886			0.824			1.000
Female	700 (48)	47 (48)		724 (48)	23 (46)		646 (48)	101 (48)		733 (48)	14 (47)	
Male	771 (52)	52 (52)		796 (52)	27 (54)		715 (52)	108 (52)		807 (52)	16 (53)	
BMI* (kg/m^2^)	25.2 ± 4.8	27.1 ± 506	**00009**	2504 ± 409	2503 ± 5.0	0.825	25.1 ± 4.8	27.0 ± 5.5	**< 0.001**	25.3 ± 4.8	27.1 ± 6.1	0.155
ASA*			**< 0.001**			**< 0.001**			**< 0.001**			0.694
I	846 (62)	26 (29)		861 (61)	11 (25)		812 (65)	60 (31)		859 (60)	13 (52)	
II	443 (33)	36 (40)		463 (33)	16 (36)		405 (32)	74 (38)		469 (33)	10 (40)	
III	66 (5)	26 (29)		78 (6)	14 (32)		35 (3)	57 (29)		90 (6)	2 (8)	
IV	2 (0)	3 (3)		2 (0)	3 (7)		1 (0)	4 (2)		5 (0)	0 (0)	
Smoker*	312 (26)	18 (21)	0.652	318 (25)	12 (30)	0.704	292 (26)	38 (22)	0.092	324 (25)	6 (22)	0.847
Diabetes	54 (4)	15 (15)	**< 0.001**	60 (4)	9 (18)	< **0.001**	44 (3)	25 (12)	**< 0.001**	67 (4)	2 (7)	0.640
Crohn’s disease	10 (1)	1 (1)	1.000	10 (1)	1 (2)	0.300	10 (1)	1 (1)	1.000	10 (1)	1 (3)	0.192
Ulcerative colitis	8 (1)	0 (0)	0.679	8 (1)	0 (0)	1.000	5 (0)	3 (1)	0.078	8 (1)	0 (0)	1.000
Preop. blood results												
WBC count (× 10^9^/l)	13.0 ± 4.4	13.8 ± 5.9	0.209	13.1 ± 4.4	14.0 ± 6.5	0.265	12.9 ± 4.3	14.4 ± 5.1	** < 0.001**	13.0 ± 4.5	14.9 ± 5.3	**0.041**
CRP (mg/l)	60 ± 71	143 ± 108	** < 0.001**	63 ± 74	149 ± 101	** < 0.001**	53 ± 63	146 ± 105	** < 0.001**	65 ± 76	109 ± 105	**0.010**
Hemoglobin (g/dl)	14.3 ± 1.5	13.4 ± 1.9	** < 0.001**	14.3 ± 1.5	13.1 ± 2.2	** < 0.001**	14.3 ± 1.5	13.7 ± 1.8	** < 0.001**	14.2 ± 1.5	14.2 ± 1.7	0.615
Creatinine (mg/dl)	0.8 ± 0.3	1.1 ± 0.5	** < 0.001**	0.9 ± 0.3	1.1 ± 0.6	**0.003**	0.8 ± 0.3	1.0 ± 0.4	** < 0.001**	0.9 ± 0.3	0.9 ± 0.2	0.700
Preop. radiological diagnostics												
App. diameter (mm)	11 ± 4	11 ± 4	0.532	11 ± 4	12 ± 6	0.379	11 ± 30	11 ± 4	0.111	11 ± 4	11 ± 4	0.418
Intraabdominal fluid	527 (36)	52 (53)	**0.001**	550 (36)	29 (58)	**0.002**	477 (35)	102 (49)	** < 0.001**	570 (37)	9 (30)	0.454

Significant p values are in bold

Data are presented as mean ± SD or *n* (%)

*LOS* length of postoperative stay, *BMI* body mass index, *ASA score (ASA)* American Society of Anesthesiologists score, *Preop.* Preoperative, *WBC* white blood cell, *App.* Appendiceal

*Incomplete data: BMI: *n* = 1137; ASA: *n* = 1448; smoker: *n* = 1306

### Preoperative, intraoperative, histopathological and postoperative findings of patients with acute appendicitis

Regarding the perioperative data following parameter were identified to be significantly associated for at least one of the outcome parameter (morbidity, major morbidity, prolonged LOS, readmission): preoperative blood results including WBC count, CRP, hemoglobin and creatinine, intraabdominal fluid in preoperative diagnostic, time to appendectomy, surgical experience, surgical approach, duration of surgery, appendix stump closure in laparoscopic surgery, necessity of coecum resection, intraoperative findings including perforation, gangrene, perithyphilitic abscess presence of complicated appendicitis, presence of complicated appendicitis or malignancy in histopathology, postoperative blood results on postoperative day 1 including WBC count, CRP, hemoglobin and creatinine, microbiological detection of bacteria as well postoperative radiological diagnostic, postoperative abdominal CT and postoperative use of antibiotics (Tables 2, 3, 4). There were no significant difference for appendiceal diameter in preoperative radiological diagnostics among the groups (Table 5).

**Table 3:** Surgical parameters

	No morbidity	Morbidity	*p* value	No major morbidity	Major morbidity	*p* value	No prolonged LOS	Prolonged LOS	*p* value	No readmission	Readmission	*p* value
	(*n* = 1471)	(*n* = 99)		(*n* = 1520)	(*n* = 50)		(≤ 5 days)	(> 5 days)		(*n* = 1540)		
							(*n* = 1361)	(*n* = 209)			(n = 30)	
Time to appendectomy			0.122			**0.002**			**0.006**			0.574
≤ 6 h	656 (45)	37 (37)		676 (45)	17 (34)		618 (45)	75 (36)		676 (44)	17 (57)	
> 6 h–≤ 12 h	426 (29)	31 (31)		442 (29)	15 (30)		382 (28)	75 (36)		450 (29)	7 (23)	
> 12 h–≤ 24 h	262 (18)	16 (16)		272 (18)	6 (12)		246 (18)	32 (15)		274 (18)	4 (13)	
> 24 h	127 (9)	15 (15)		130 (9)	12 (24)		115 (8)	27 (13)		140 (9)	2 (7)	
Surgical experience			0.079			0.077			**0.001**			0.687
Resident	406 (28)	19 (19)		417 (27)	8 (16)		388 (29)	37 (18)		418 (27)	7 (23)	
Specialist	1065 (72)	80 (81)		1103 (73)	42 (84)		973 (71)	172 (82)		1122 (73)	23 (77)	
Surgical approach			** < 0.001**			** < 0.001**			** < 0.001**			** < 0.001**
Laparoscopic	1104 (75)	49 (50)		1129 (74)	24 (48)		1051 (77)	102 (49)		1136 (74)	17 (57)	
Open	316 (21)	34 (34)		332 (22)	17 (36)		286 (21)	64 (31)		343 (22)	7 (23)	
Conversion	51 (4)	16 (16)		59 (4)	8 (16)		24 (2)	43 (21)		61 (4)	6 (20)	
Duration of surgery (min)	66 ± 27	90 ± 40	** < 0.001**	66 ± 28	90 ± 47	** < 0.001**	64 ± 25	90 ± 39	** < 0.001**	67 ± 28	81 ± 46	0.074
Appendix stump closure (in laparoscopic surgery)			0.235			0.542			**0.036**			0.241
Stapler	920 (83)	45 (92)		943 (84)	22 (92)		872 (83)	93 (91)		948 (84)	17 (100)	
Endoloop	172 (16)	4 (8)		174 (15)	2 (8)		169 (16)	7 (7)		176 (16)	0 (0)	
Other	12 (1)	0 (0)		12 (1)	0 (0)		10 (1)	2 (2)		12 (1)	0 (0)	
Coecum resection	57 (4)	12 (12)	**0.001**	64 (4)	5 (10)	0.065	38 (3)	31 (15)	** < 0.001**	67 (4)	2 (7)	0.640
Intraoperative findings												
Perforation	280 (19)	60 (61)	** < 0.001**	309 (20)	31 (62)	** < 0.001**	207 (15)	133 (64)	** < 0.001**	333 (22)	7 (23)	0.823
Necrosis or gangrene	106 (7)	23 (23)	** < 0.001**	118 (8)	11 (22)	**0.002**	82 (6)	47 (23)	** < 0.001**	128 (8)	1 (3)	0.507
Perithyphilitic abscess	128 (9)	33 (33)	** < 0.001**	146 (10)	15 (30)	** < 0.001**	86 (6)	75 (36)	** < 0.001**	154 (10)	7 (23)	**0.028**
Complicated appendicitis	330 (22)	66 (67)	** < 0.001**	362 (24)	34 (68)	** < 0.001**	244 (18)	152 (73)	** < 0.001**	383 (25)	13 (43)	**0.031**

Significant p values are in bold

Data are presented as mean ± SD or *n* (%)

*LOS* length of postoperative stay

**Table 4:** Histopathological and postoperative parameters

	No morbidity	Morbidity	*p* value	No major morbidity	Major morbidity	*p* value	No prolonged LOS	Prolonged LOS	*p* value	No readmission	Readmission	*p* value
	(*n* = 1471)	(*n* = 99)		(*n* = 1520)	(*n* = 50)		(≤ 5 days)	(> 5 days)		(*n* = 1540)		
							(*n* = 1361)	(*n* = 209)			(*n* = 30)	
Histopathological findings												
Complicated appendicitis	500 (34)	67 (68)	** < 0.001**	531 (35)	36 (74)	** < 0.001**	425 (31)	142 (68)	** < 0.001**	555 (36)	12 (40)	0.703
Malignancy	20 (1)	1 (1)	1.000	20 (1)	1 (2)	1.000	15 (1)	6 (3)	**0.049**	16 (1)	5 (17)	** < 0.001**
Blood results on POD 1*												
WBC count (× 10^9^/l)	9.2 ± 3.2	12.2 ± 7.0	** < 0.001**	9.3 ± 3.4	12.1 ± 7.7	**0.010**	9.0 ± 3.0	12.0 ± 5.5	** < 0.001**	9.4 ± 3.6	11.7 ± 5.4	**0.003**
CRP (mg/l)	114 ± 86	217 ± 98	** < 0.001**	117 ± 88	222 ± 103	** < 0.001**	103 ± 78	227 ± 90	** < 0.001**	120 ± 90	177 ± 101	**0.002**
Hemoglobin (g/dl)	12.6 ± 1.5	11.7 ± 1.9	** < 0.001**	12.6 ± 1.5	11.1 ± 2.1	** < 0.001**	12.7 ± 1.5	12.0 ± 1.8	** < 0.001**	12.6 ± 1.5	12.1 ± 1.8	0.118
Creatinine (mg/dl)	0.8 ± 0.3	1.0 ± 0.5	** < 0.001**	0.8 ± 0.3	1.0 ± 0.5	**0.023**	0.8 ± 0.3	0.9 ± 0.4	0.152	0.8 ± 0.3	0.8 ± 0.2	0.731
Microbiol. detection of bacteria**	200 (48)	31 (91)	** < 0.001**	214 (49)	17 (90)	**0.001**	159 (42)	72 (94)	** < 0.001**	225 (50)	6 (67)	0.504
Postop. any radiol. diagnostic necessary	2 (0)	53 (54)	** < 0.001**	19 (1)	36 (72)	** < 0.001**	3 (0)	52 (25)	** < 0.001**	54 (4)	1 (3)	1.000
Postop. abdominal CT	1 (0)	35 (35)	** < 0.001**	6 (0)	30 (60)	** < 0.001**	1 (0)	35 (17)	** < 0.001**	36 (2)	0 (0)	0.645
Postop. antibiotics	747 (51)	86 (87)	** < 0.001**	793 (52)	40 (80)	** < 0.001**	646 (48)	187 (90)	** < 0.001**	815 (53)	18 (60)	0.467

Significant p values are in bold

Data are presented as mean [range] or *n* (%)

*LOS* length of postoperative stay, *WBC* white blood cell, *Postop.* Postoperative, *radiol.* Radiological, *POD* postoperative day, *Microbiol.* Microbiological

*Postoperative blood examination not done in all patients: *n* = 1388

**Microbiological examination in 455 (29%) patients done

**Table 5:** Multivariate analysis of risk factors for morbidity, major morbidity, prolonged postoperative hospital stay and readmission

	Morbidity			Major morbidity			Prolonged LOS			Readmission		
	OR	CI	*p* value	OR	CI	*p* value	OR	CI	*p* value	OR	CI	*p* value
Age (high vs. low)*	**3.79**	**2.22–6.49**	** < 0.001**	**2.90**	**1.37–6.10**	**0.005**	**2.41**	**1.55–3.75**	** < 0.001**	0.72	0.29–1.81	0.485
Gender (male vs. female)	0.81	0.46–1.44	0.482	0.64	0.30–1.37	0.249	0.73	0.44–1.22	0.231	0.96	0.39–2.36	0.955
Diabetes (yes vs. no)	1.44	0.67–3.09	0.355	1.66	0.63–4.42	0.309	1.42	0.61–3.31	0.411	1.42	0.30–6.75	0.662
Preop. WBC count (high vs. low)**	**2.01**	**1.22–3.30**	**0.006**	1.82	0.91–3.63	0.091	**2.19**	**1.43–3.36**	** < 0.001**	**2.28**	**1.01–5.18**	**0.049**
Preop. CRP (high vs. low)***	**2.45**	**1.40–4.28**	**0.002**	**2.56**	**1.16–5.65**	**0.020**	**2.23**	**1.42–3.52**	** < 0.001**	2.04	0.78–5.36	0.149
Preop. hemoglobin (high vs. low)****	**0.45**	**0.25–0.78**	**0.005**	**0.28**	**0.13–0.61**	**0.001**	**0.57**	**0.34–0.94**	**0.029**	6.48	0.92–37.53	0.067
Preop. creatinine (high vs. low)*****	1.74	0.98–3.11	0.061	1.66	0.76–3.63	0.205	0.98	0.52–1.85	0.958	1.17	0.44–3.10	0.756
Intraabdominal fluid in radiological diagnostic (yes vs. no)	1.28	0.78–2.09	0.329	1.61	0.83–3.12	0.159	0.98	0.64–1.52	0.943	0.67	0.28–1.61	0.373
Time to appendectomy (high vs. low)******	**1.90**	**1.17–3.08**	**0.009**	**2.48**	**1.26–4.85**	**0.008**	1.52	0.98–2.35	0.060	4.55	0.87–23.32	0.069
Surgical experience (resident vs. specialist)	0.97	0.53–1.77	0.914	1.19	0.50–2.82	0.695	1.15	0.68–1.93	0.610	0.95	0.37–2.43	0.906
Surgical approach			0.373			0.773			** < 0.001**			0.187
Open vs. laparoscopic	1.37	0.79–2.39	0.263	1.21	0.57–2.57	0.617	1.41	0.86–2.32	0.176	1.27	0.48–3.33	0.632
Conversion vs. laparoscopic	1.57	0.72–3.42	0.262	1.43	0.48–4.30	0.525	**4.91**	**2.39–10.07**	** < 0.001**	3.24	0.92–11.37	0.067
Duration of surgery (high vs. low)*******	**2.67**	**1.53–4.68**	** < 0.001**	1.27	0.63–2.59	0.506	**1.90**	**1.21–2.98**	**0.005**	2.07	0.87–4.92	0.100
Coecum resection (yes vs. no)	0.74	0.32–1.70	0.472	0.47	0.14–1.57	0.219	1.54	0.74–3.18	0.245	0.65	0.12–3.53	0.622
Intraoperative complicated app (yes vs. no)	1.70	0.94–3.07	0.079	1.63	0.72–3.66	0.238	**4.00**	**2.39–6.70**	** < 0.001**	1.30	0.42–4.01	0.649
Histopathological complicated app (yes vs. no)	1.33	0.76–2.31	0.316	1.86	0.86–4.06	0.117	1.06	0.66–1.71	0.812	0.62	0.23–1.68	0.345
Malignancy (yes vs. no)	0.29	0.04–2.44	0.257	0.83	0.10–7.00	0.864	1.43	0.42–4.81	0.565	**15.27**	**4.47–52.12**	** < 0.001**
Morbidity (yes vs. no)							**23.38**	**12.16–44.94**	** < 0.001**	0.69	0.16–3.02	0.626
Prolonged LOS (yes vs. no)										1.50	0.46–4.93	0.504

Significant p values are in bold

Appendiceal stump closure (only laparoscopic appendectomies) as well as BMI, ASA, postoperative blood results and microbiological detection of bacteria (incomplete data) were excluded

*LOS* length of postoperative stay, *Preop.* Preoperative, *WBC* white blood cell, *app.* appendicitis

*Cut-offs for age assessed by ROC analysis (Supplementary Table 1): Morbidity: 50 years, Major morbidity: 52 years, Prolonged LOS: 50 years, Readmission: 29 years

**Cut-offs for WBC count assessed by ROC analysis (Supplementary Table 1): Morbidity: 14.7 × 10^9^/l, Major morbidity: 15.2 × 10^9^/l, Prolonged LOS: 13.8 × 10^9^/l, Readmission: 13.9 × 10^9^/l

***Cut-offs for CRP assessed by ROC analysis (Supplementary Table 1): Morbidity: 71 mg/l, Major morbidity: 74 mg/l, Prolonged LOS: 83 mg/l, Readmission: 70 mg/l

****Cut-offs for hemoglobin assessed by ROC analysis (Supplementary Table 1): Morbidity: 13.7 g/dl, Major morbidity: 13.7 g/dl, Prolonged LOS: 13.7 g/dl, Readmission: 17.4 g/dl

*****Cut-offs for creatinine assessed by ROC analysis (Supplementary Table 1): Morbidity: 1.0 mg/dl, Major morbidity: 1.0 mg/dl, Prolonged LOS: 1,1 mg/dl, Readmission: 1.0 mg/dl

******Cut-offs for time to appendectomy assessed by ROC analysis (Supplementary Table 1): Morbidity: 442 min, Major morbidity: 442 min, Prolonged LOS: 345 min, Readmission: 2846 min

*******Cut-offs for duration of surgery assessed by ROC analysis (Supplementary Table 1): Morbidity: 64 min, Major morbidity: 81 min, Prolonged LOS: 64 min, Readmission: 75 min

### Multivariate analysis of patients with acute appendicitis

Sixteen (morbidity and major morbidity) respectively seventeen (prolonged LOS) respectively eighteen (readmission) identified risk factors were included to multivariate analysis (Table 5), whereby the cutoffs of the metric risk factors were previously determined using ROC analysis (Supplementary Table 1):

**Table 6:** Risk for morbidity, major morbidity, prolonged hospital stay and readmission depending on the number of independent pre- and intraoperative risk factors

Number of risk factors	Morbidity*		Major morbidity**		Prolonged hospital stay***		Readmission****	
	*n*	Risk	*n*	Risk	*n*	Risk	*n*	Risk
All patients	1570		1570		1570		1570	
0	188	0.0%	410	0.3%	237	1.1%	1550	1.1%
1	363	0.6%	546	0.7%	438	0.9%	17	3.3%
2	437	3.0%	365	3.3%	393	4.3%	3	33.3%
3	334	6.6%	201	9.5%	205	11.2%		
4	177	16.4%	46	23.9%	147	39.5%		
5	62	37.1%			83	50.6%		
6	9	77.8%			49	79.6%		
7					16	87.5%		
8					2	100%		

*Six independent risk factors for morbidity: age > 50 years, preoperative WBC count > 14.7 × 10^9^/l, preoperative CRP > 71 mg/l, preoperative hemoglobin ≤ 13.7 g/dl, time to appendectomy > 442 min, duration of surgery > 64 min. Cut-offs for metric parameters assessed by ROC analysis (Supplementary Table 1)

**Four independent risk factors for major morbidity: age > 52 years, preoperative CRP > 74 mg/l, preoperative hemoglobin ≤ 13.7 g/dl, time to appendectomy > 442 min. Cut-offs for metric parameters assessed by ROC analysis (Supplementary Table 1)

***Eight independent risk factors for a prolonged hospital stay: age > 50 years, preoperative white blood cells > 13.8 (× 10^9^/l), preoperative CRP > 83 mg/l, preoperative hemoglobin ≤ 13.7 g/dl, conversion, duration of surgery > 64 min, intraoperative complicated appendicitis, morbidity. Cut-offs for metric parameters assessed by ROC analysis (Supplementary Table 1)

****Two independent risk factors for readmission: preoperative WBC count > 13.9 × 10^9^/l, malignancy. Cut-offs for metric parameters assessed by ROC analysis (Supplementary Table 1)

There were six independent risk factors for morbidity: age > 50 years (OR 3.79 (2.22–6.49); *p* < 0.001), preoperative WBC count > 14.7 × 10^9^/l (OR 2.01 (1.22–3.30); *p* = 0,006), preoperative CRP > 71 mg/l (OR 2.45 (1.40–4.28); *p* = 0.002), preoperative hemoglobin ≤ 13.7 g/dl (OR 0.45 (0.25–0.78); *p* = 0,005), time to appendectomy > 442 min (OR 1.90 (1.17–3.08); *p* = 0.009) and duration of surgery > 64 min (OR 2.67 (1.53–4.68); *p* < 0.001).

Four independent risk factors could be identified for major morbidity: age > 52 years (OR 2.90 (1.37–6.10); *p* = 0.005), preoperative CRP > 74 mg/l (OR 2.56 (1.16–5.65); *p* = 0.020), preoperative hemoglobin ≤ 13.7 g/dl (OR 0.28 (0.13–0.61); *p* = 0.001) and time to appendectomy > 442 min (OR 2.48 (1.26–4.85); *p* = 0.008).

Eight parameters were independent risk factors for a prolonged hospital stay: age > 50 years (OR 2.41 (1.55–3.75); *p* < 0.001), preoperative WBC count > 13.8 × 10^9^/l (OR 2.19 (1.43–3.36); *p* < 0.001), preoperative CRP > 83 mg/l (OR 2.23 (1.42–3.52); *p* < 0.001), preoperative hemoglobin ≤ 13.7 g/dl (OR 0.57 (0.34–0.94); *p* = 0.029), need for conversion (OR 4.91 (2.39–10.07); *p* < 0.001), surgery duration > 64 min (OR 1.90 (1.21–2.98); *p* = 0.005), presence of intraoperative complicated appendicitis (OR 4.00 (2.39–6.70; *p* < 0.001) and presence of postoperative morbidity (OR 23.38 (12.16–44.94); *p* < 0.001).

Multivariate analysis revealed two independent risk factors for readmission: preoperative WBC count > 13.9 × 10^9^/l (OR 2.28 (1.01–5.18); *p* = 0.049) and presence of malignancy (OR 15.27 (4.47–52.12); *p* < 0.001).

### Absolute risk values for morbidity, major morbidity, prolonged LOS and readmission according to presence of the identified independent risk factors in patients with acute appendicitis

Table 6 shows the absolute risk values for morbidity, major morbidity, prolonged LOS and readmission according to the number of present identified independent risk factors**.**

## Discussion

Appendectomy for suspected acute appendicitis is one of the most common emergency surgeries and is usually associated with moderate morbidity. However, particularly in the context of the ongoing discussion about non-operative management of acute appendicitis, advocates of the conservative therapy use negative appendectomies and especially surgical morbidity as an argument. The aim of the present study was to analyze risk factors associated with the development of morbidity and major morbidity, with a prolonged length of hospital stay and with the need of readmission in patients undergoing appendectomy due to acute appendicitis.

In our cohort, the rate of negative appendectomies was low (4%), which underlines a differentiated indication for surgery. In literature, rates of negative appendectomy vary enormously and are usually higher than ours [16–18]. Negative appendectomies were associated with a shorter hospital stay compared to appendectomies for acute appendicitis and with a low morbidity (3%) in our study. These results are consistent with previous data from Lee et al., although a longer LOS was only observed comparing complicated appendicitis and negative appendectomy [18].

The overall complication rate after appendectomy for acute appendicitis in our study was 6% and therefore consistent with previous data from literature [10–14]. We could identify six independent risk factors for morbidity: higher age, higher preoperative WBC count, higher preoperative CRP, lower preoperative hemoglobin, longer time to surgery and longer duration of surgery. All these associations are supported by previous studies [11, 19–22]. Particularly high WBC counts and high CRP values as well as indirectly a longer surgical time are indicators of severe appendicitis, which per se are likely to be associated with higher morbidity. In literature, higher creatinine levels at admission could be identified as further risk factor, but preoperative creatinine failed to be significant in our results [11, 21].

In our cohort, about half of patients with postoperative complications had major morbidity (Clavien–Dindo III–V). This rate was slightly higher than those reported in literature [11, 12, 14, 21, 23]. On the other side, mortality was 0.13% in our study group, which is lower than reported by two big European analysis with over 100,000 patients (mean mortality of 2.1–2.4 per 1000 patients) [24, 25]. Our results revealed higher age, higher preoperative CRP, lower preoperative hemoglobin and longer time to surgery as independent risk factors for major morbidity. Comparable data for the outcome of major morbidity are limited. A polish analysis with over 4000 laparoscopic appendectomies showed an association between complicated appendicitis and intraoperative adverse events with major morbidity, which could not be confirmed by our data [14].

Our data support the importance of timely surgery after diagnosis to reduce the risk of morbidity and major morbidity. The best timing of surgery for acute appendicitis is a frequently discussed aspect—especially in the face of limited surgical capacity and different competing emergency surgeries. Previous studies show controversial results [11, 26–33]. A Dutch meta-analysis from 2018 show that delaying appendectomy up to 24 h after admission does not appear to be a risk factor for complicated appendicitis, postoperative surgical-site infection or morbidity, when an uncomplicated appendicitis is presumed at admission [34]. Li et al. demonstrated no significant difference in complicated appendicitis incidence by delayed appendectomy over 12 h; however the risk of wound infection was slightly increased [35]. Moreover, the risk of complicated appendicitis was more strongly associated with overall elapsed time from symptoms presentation [35]. However, our study did not include the delay from symptoms presentation until the incision time. In our collective, the ROC analysis showed a cut-off of 442 min as the best differentiation for the presence of an increased risk of morbidity after appendectomy for acute appendicitis. A similar cut-off were observed in another study by Teixeira et al., that showed an increased incidence of wound infections and intra-abdominal abscess after a delay of more than 6 h for performing appendectomy [32]. According to a subgroup analysis in our collective, early surgery is particularly important in the presence of complicated appendicitis, which is in line with existing data [11].

Our data confirm that occurrence of morbidity and major-morbidity result in further sequelae for the affected patients—especially further radiological diagnostics and higher need of postoperative antibiotics. However, based on these associations, our data cannot provide further evidence to an ongoing debated issue of the need of postoperative antibiotics. A recent Dutch meta-analysis found no clear evidence about the duration of antibiotic therapy and the incidence of intra-abdominal abscesses, which underlines the need for further high-quality studies to make adequate recommendations on this topic [36].

In our study, the occurrence of a complication also represented the most important risk factor for a longer hospital stay. Patients with postoperative complications had a 23-fold increased risk of prolonged hospital stay. In addition, other risk factors could also be identified—most of them were again indicator of severe appendicitis: age, preoperative laboratories (CRP, WBC count and hemoglobin) as well as conversions rate, duration of surgery and presence of an intraoperative complicated appendicitis. These results are in line with previous studies, which could reveal a correlation between complicated appendicitis, age, postoperative morbidity and converted appendectomy with more extended hospital stay [14, 37–41]. Moreover, Zhang et al. demonstrated an association between duration of surgery, preoperative WBC count, and CRP levels with a prolonged hospital stay [40].

Our readmission rate was low (2%) compared to a current meta-analysis (readmission rate of 4.8%) [42]. Our identified risk factors for readmission were high preoperative WBC count and malignancy. Malignancy requires a high probability of further therapy, which explains a renewed hospitalization. The association between high WBC counts and readmission may be explained again by the causal chain of more severe appendicitis and the resulting higher morbidity. However, the already mentioned meta-analysis including over 800,000 appendectomies identified the presence of complicated appendicitis and diabetes mellitus as risk factors, which was not confirmed in our study [42].

As a consequence, the identified risk factors of the surgical outcome parameters morbidity, major morbidity, length of hospital stay and readmission can be used to take early preventive measures in patients at risk and to identify complications early on through intensified postoperative monitoring to alleviate the extent of the complication. However, the risk factors identified are less suitable for the indication for surgery, since only preoperative risk parameters can be used and some of the preoperative risk factors (age, high CRP values) represent also risk factors for the presence of appendix malignancy [43].

Several limitations exist regarding our data: first, the retrospective nature of this study and single-center design can incur some bias. Second, there is a selections bias, as patients with signs of complicated appendicitis were operated more quickly and by more experienced surgeons. Moreover, inclusion of open and minimal-invasive approach for appendectomy could bias results, but has the advantage to reflect a real-world scenario. Third, there is a lack of established protocols used in the University Hospital Erlangen to treat patients with acute appendicitis potentially affecting the results of our study. Fourth, there are always some interferences between parameters, e.g. duration of surgery and conversion rate depends on experience of surgeons.

## Conclusion

Among patients undergoing appendectomy for acute appendicitis, accurate risk assessment can help to identify patients with a higher risk for worse surgical outcome. Especially modifiable risk factors, such as time to appendectomy and preoperative hemoglobin, should be addressed. Anyway, risk classification can be useful as patients at risk may benefit from special attention in the postoperative course and from a more customized therapy.

## Supplementary Information

Below is the link to the electronic supplementary material.

Supplementary file1 (DOCX 20 KB)

## Data Availability

All relevant data supporting the conclusions are included within the manuscript, the tables and the supplemental table.

## References

[1] Ferris M, Quan S, Kaplan BS (2017). The global incidence of appendicitis. Ann Surg.

[2] Stöß C, Nitsche U, Neumann PA (2021). Acute appendicitis: trends in surgical treatment—a population-based study of over 800 000 patients. Deutsches Aerzteblatt International.

[3] Varadhan KK, Neal KR, Lobo DN (2012). Safety and efficacy of antibiotics compared with appendicectomy for treatment of uncomplicated acute appendicitis: meta-analysis of randomised controlled trials. BMJ.

[4] Hansson J, Körner U, Khorram-Manesh A (2009). Randomized clinical trial of antibiotic therapy versus appendicectomy as primary treatment of acute appendicitis in unselected patients. Br J Surg.

[5] McCutcheon BA, Chang DC, Marcus LP (2014). Long-term outcomes of patients with nonsurgically managed uncomplicated appendicitis. J Am Coll Surg.

[6] Vons C, Barry C, Maitre S, Pautrat K (2011). Amoxicillin plus clavulanic acid versus appendicectomy for treatment of acute uncomplicated appendicitis: an open-label, non-inferiority, randomised controlled trial. Lancet.

[7] Salminen P, Paajanen H, Rautio T (2015). Antibiotic therapy vs appendectomy for treatment of uncomplicated acute appendicitis: the APPAC randomized clinical trial. JAMA.

[8] Birnbaum BA, Wilson SR (2000). Appendicitis at the millennium. Radiology.

[9] Wilms IM, de Hoog DE, de Visser DC, Janzing HM (2011). Appendectomy versus antibiotic treatment for acute appendicitis. Cochrane Database Syst Rev.

[10] Angeramo CA, Dreifuss NH, Olivero AA (2020). Risk factors for readmission after short-hospital-stay laparoscopic appendectomy. World J Surg.

[11] Andert A, Alizai HP, Klink CD (2017). Risk factors for morbidity after appendectomy. Langenbeck’s Arch Surg.

[12] Kostov K (2021). Risk factors and outcomes for septic complications after laparoscopic appendectomy. J IMAB Ann Proc (Sci Papers).

[13] Minutolo V, Licciardello A, di Stefano B (2014). Outcomes and cost analysis of laparoscopic versus open appendectomy for treatment of acute appendicitis: 4-years experience in a district hospital. BMC Surg.

[14] Walędziak M, Lasek A, Wysocki M (2019). Risk factors for serious Morbidity, prolonged length of stay and hospital readmission after laparoscopic appendectomy—results from Pol-LA (Polish Laparoscopic Appendectomy) multicenter large cohort study. Sci Rep.

[15] Dindo D, Demartines N, Clavien PA (2004). Classification of surgical complications: a new proposal with evaluation in a cohort of 6336 patients and results of a survey. Ann Surg.

[16] Seetahal SA, Bolorunduro OB, Sookdeo TC, Oyetunji TA, Greene WR, Frederick W, Cornwell EE, Chang DC, Siram SM (2011). Negative appendectomy: a 10-year review of a nationally representative sample. Am J Surg.

[17] Tamini N, Santurro L, Chiappetta MF, Gattuso I, Barbieri C, Fattori L, Gianotti L (2020). Morbidity after negative appendectomy: a single-centre experience on 627 cases. Eur J Trauma Emerg Surg.

[18] Lee M, Paavana T, Mazari F, Wilson TR (2014). The morbidity of negative appendicectomy. Ann R Coll Surg Engl.

[19] Patel SV, Nanji S, Brogly SB (2018). High complication rate among patients undergoing appendectomy in Ontario: a population-based retrospective cohort study. Can J Surg.

[20] Pereira BM, Mendes CA, Ruano RM (2019). Acute appendicitis may no longer be a predominant disease of the young population. Anaesthesiol Intensive Therapy.

[21] Kim JY, Kim JW, Park JH (2019). Early versus late surgical management for complicated appendicitis in adults: a multicenter propensity score matching study. Ann Surg Treat Res.

[22] de Wijkerslooth EML, de Jonge J, van den Boom AL (2019). Postoperative outcomes of patients with nonperforated gangrenous appendicitis: a national multicenter prospective cohort analysis. Dis Colon Rectum.

[23] Sood A, Meyer CP, Abdollah F (2017). Minimally invasive surgery and its impact on 30-day postoperative complications, unplanned readmissions and mortality. Br J Surg.

[24] Kotaluoto S, Ukkonen M, Pauniaho SL (2017). Mortality related to appendectomy; a population based analysis over two decades in Finland. World J Surg.

[25] Blomqvist PG, Andersson RE, Granath F (2001). Mortality after appendectomy in Sweden, 1987–1996. Ann Surg.

[26] Abou-Nukta F, Bakhos C, Arroyo K (2006). Effects of delaying appendectomy for acute appendicitis for 12 to 24 hours. Arch Surg.

[27] Bhangu A, United Kingdom National Surgical Research Collaborative (2014). Safety of short, in-hospital delays before surgery for acute appendicitis: multicentre cohort study, systematic review, and meta-analysis. Ann Surg.

[28] Kim HK, Kim YS, Lee SH (2017). Impact of a delayed laparoscopic appendectomy on the risk of complications in acute appendicitis: a retrospective study of 4,065 patients. Dig Surg.

[29] Fair BA, Kubasiak JC, Janssen I (2015). The impact of operative timing on outcomes of appendicitis: a National Surgical Quality Improvement Project analysis. Am J Surg.

[30] March B, Gillies D, Gani J (2014). Appendicectomies performed >48 hours after admission to a dedicated acute general surgical unit. Ann R Coll Surg Engl.

[31] Teixeira PG, Sivrikoz E, Inaba K (2012). Appendectomy timing: waiting until the next morning increases the risk of surgical site infections. Ann Surg.

[32] Giraudo G, Baracchi F, Pellegrino L (2013). Prompt or delayed appendectomy? Influence of timing of surgery for acute appendicitis. Surg Today.

[33] Andert A, Alizai HP, Klink CD (2017). Risk factors for morbidity after appendectomy. Langenbecks Arch Surg.

[34] van Dijk ST, van Dijk AH, Dijkgraaf MG, Boermeester MA (2018). Meta-analysis of in-hospital delay before surgery as a risk factor for complications in patients with acute appendicitis. Br J Surg.

[35] Li J, Xu R, Hu DM, Zhang Y, Gong TP, Wu XL (2019). Effect of delay to operation on outcomes in patients with acute appendicitis: a systematic review and meta-analysis. J Gastrointest Surg.

[36] van den Boom AL, de Wijkerslooth EML, Wijnhoven BPL (2020). Systematic review and meta-analysis of postoperative antibiotics for patients with a complex appendicitis. Dig Surg.

[37] Pedziwiatr M, Lasek A, Wysocki M (2019). Complicated appendicitis: risk factors and outcomes of laparoscopic appendectomy—polish laparoscopic appendectomy results from a multicenter, large-cohort study. Ulus Travma ve Acil Cerrahi Derg.

[38] Ward NT, Ramamoorthy SL, Chang DC (2014). Laparoscopic appendectomy is safer than open appendectomy in an elderly population. J Soc Laparoendoscopic Surgeons..

[39] Moreira LF, Garbin HI, Da-Natividade GR (2018). Predicting factors of postoperative complications in appendectomies. Rev Col Bras Cir.

[40] Zhang P, Zhang Q, Zhao H (2020). Factors affecting the length of hospital stay after laparoscopic appendectomy: a single center study. PLoS ONE.

[41] Demir C, Çelik Y, Gider Ö (2007). The factors affecting length of stay of the patients undergoing appendectomy surgery in a military teaching hospital. Military Med.

[42] Bailey K, Choynowski M, Kabir SMU (2019). Meta-analysis of unplanned readmission to hospital post-appendectomy: an opportunity for a new benchmark. ANZ J Surg.

[43] Brunner M, Lapins P, Langheinrich M (2020). Risk factors for appendiceal neoplasm and malignancy among patients with acute appendicitis. Int J Colorectal Dis.

