# Supplementary material for: Risk factors for postoperative morbidity, prolonged length of stay and hospital readmission after appendectomy for acute appendicitis
Source: Eur J Trauma Emerg Surg. 2023 Jan 28;49(3):1355–66. doi: 10.1007/s00068-023-02225-9 (PMC10229676; doi:10.1007/s00068-023-02225-9)
Supplement: Supplementary file 1 — Supplementary file1 (DOCX 20 KB) [file 68_2023_2225_MOESM1_ESM.docx]

**Sup. Tab. 1:** Cut-off analysis for metric independent risk factors for morbidity, major morbidity, prolonged hospital stay and readmission

|  | Morbidity | | Major morbidity | | Prolonged hospital stay | | Readmission | |
| --- | --- | --- | --- | --- | --- | --- | --- | --- |
| Metric risk factors | Best Cut-off | ROC analysis:  Sensitivity + Specificity | Best Cut-off | ROC analysis:  Sensitivity + Specificity | Best Cut-off | ROC analysis:  Sensitivity + Specificity | Best  Cut-off | ROC analysis:  Sensitivity + Specificity |
| Age (years) | 50 | 1,473 | 52 | 1,459 | 50 | 1,424 | 29 | 1,126 |
| Preop. WBC count (x10^9^/l) | 14,7 | 1,114 | 15,2 | 1,137 | 13,8 | 1,149 | 13,9 | 1,185 |
| Preop. CRP (mg/l) | 71 | 1,450 | 74 | 1,485 | 83 | 1,461 | 70 | 1,291 |
| Preop. hemoglobin (g/dl) | 13,7 | 1,218 | 13,7 | 1,303 | 13,7 | 1,172 | 17,4 | 1,063 |
| Preop. creatinine (mg/dl) | 1,0 | 1,249 | 1,0 | 1,288 | 1,1 | 1,157 | 1,0 | 1,098 |
| Time to appendectomy (min) | 442 | 1,119 | 442 | 1,189 | 345 | 1,111 | 2846 | 1,048 |
| Duration of surgery (min) | 64 | 1,336 | 81 | 1,274 | 64 | 1,349 | 75 | 1,258 |

Preop. = preoperative; WBC = white blood cell.
